# Prostaglandins E2 signal mediated by receptor subtype EP2 promotes IgE production in vivo and contributes to asthma development

**DOI:** 10.1038/srep20505

**Published:** 2016-02-08

**Authors:** Yuhan Gao, Chunyan Zhao, Wei Wang, Rong Jin, Qian Li, Qing Ge, Youfei Guan, Yu Zhang

**Affiliations:** 1 Department of Immunology, and Key Laboratory of Medical Immunology of Ministry of Public Health, Peking University Health Science Center, Beijing, China; 2 Advanced Institute for Medical Sciences, Dalian Medical University, Dalian, China; 3 State Key Laboratory of Natural and Biomimetic Drugs, Peking University, Beijing, China

## Abstract

Prostaglandins E2 (PGE2) has been shown to enhance IgE production by B cells *in vitro*. The physiological and pathological relevance of this phenomenon and the underlying molecular mechanism, however, remain to be elucidated. B cells from wild type and EP2-deficient mice were compared in culture for their responses to PGE2 in terms of IgE class switching and production. Ovalbumin (OVA)-induced asthma models were used to evaluate the impact of EP2-deficiency on IgE responses and the development of asthma. PGE2 promoted IgE class switching, generation of IgE^+^ cells and secretion of IgE by B cells stimulated with LPS+IL4. These effects were much attenuated as a consequence of EP2 deficiency. Consistent with the *in vitro* data, EP2-deficient mice showed a markedly suppressed IgE antibody response and developed less pronounced airway inflammation in the OVA-induced asthma model. Mechanistic studies demonstrated that PGE2, in an EP2-depedent manner, enhanced STAT6 activation induced by IL-4, thereby promoting the expression of IgE germline and post switch transcripts and the transcription of activation-induced cytidine deaminase (AID). Collectively, these data support an important regulatory role of the PGE2-EP2-STAT6 signaling pathway in IgE response and allergic diseases.

Prostaglandins E2 (PGE2) is the most abundantly produced prostanoid in the body. Its production begins with the liberation of arachidonic acid from membrane phospholipids by phospholipase A2. Arachidonic acid is then converted into prostaglandin H2 (PGH2) by cyclooxygenases (COX). The metabolism of PGH2 to PGE2 is catalyzed by prostaglandin E synthase (PGES). While COX-1, cytosolic PGES and microsomal PGES-2 (mPGES-2) are constitutively expressed, COX-2 and mPGES-1 are induced in response to various pro-inflammatory and mitogenic stimuli^1^,^2^.

PGE2 functions by acting on one of the four E prostanoid (EP) receptors, EP1-4, all belonging to the rhodopsin-type G-protein-coupled membrane receptor family^2^,^3^. These subtypes of EP receptor differ in the intracellular signaling. EP1 receptor is linked to the Gq protein and its engagement leads to phospholipase C activation and an increase in intracellular calcium. Both EP2 and EP4 receptors are coupled to the Gs protein and primarily signal through the adenylate cyclase-triggered cAMP-PKA-CREB pathway. In comparison to the rapid desensitization of EP4 upon interaction with PGE2, EP2 seems to be more resistant to ligand-induced desensitization^4^. Moreover, EP4 is featured by the ability to activate phosphatidylinositol 3 kinase (PI3K) signaling pathways^5^. On the other hand, the signal mediated by the Gi protein downstream of EP3 usually leads to inhibition of adenylate cyclase and reduction in intracellular cAMP levels. The differential expression of these receptors determines the specific physiological response in different cell types.

PGE2 displays pleiotropic actions in a wide array of tissues, ranging from the cardiovascular, neural, renal, respiratory, hematopoietic, to the immune system^6^,^7^,^8^,^9^,^10^,^11^,^12^. As increasing studies indicated an important role of PGE2 in a number of inflammatory disorders, its immunomodulatory function has drawn a lot of attention in recent years. PGE2 is generally considered being a suppressor of T cell activation and proliferation. Moreover, PGE2 is believed to have a profound influence on the pattern of CD4^+^ T cell responses. By down-regulating IL-12 expression in antigen presenting cells (APC), it creates a cytokine milieu which favors the development of Th2 cells while suppressing Th1 differentiation^13^,^14^. This prevailing view, however, is challenged by recent studies with mice deficient for EP2/EP4 receptors^15^,^16^. Yao *et al.* have demonstrated that EP2/EP4-mediated activation of the cAMP-PKA pathway actually facilitates IL-12-driven Th1 differentiation. PGE2 is also involved in the regulation of Th17 differentiation. On one hand, PGE2 potently suppresses the development of Th17 cells from naïve T cells induced by IL-6 and TGF-β^17^. On the other hand, it signals through EP2/EP4 to amplify IL-23-mediated Th17 cell expansion^15^,^18^. In comparison to T cells, limited information is available about PGE2-mediated action on B cells. PGE2 is reported to inhibit certain events of B cell activation such as cellular enlargement and up-regulation of class II major histocompatibility complex (MHC) and FcεRII (a low affinity IgE receptor) expression^19^,^20^. On the contrary, a much increased quantity of IgE was produced by LPS+IL-4-stimulated B cells in the presence of PGE2^19^,^20^,^21^,^22^.

The commitment of a B cell to an IgE-producing cell is critically dependent on a unique type of intrachromosomal deletional recombination called class switch recombination (CSR)^23^. Two main pathways of CSR have been described for IgE: a direct pathway from the IgM to the IgE isotype and a sequential pathway from IgM to an IgG1 intermediate and then to IgE^24^,^25^,^26^. A functional IgE gene is thus generated by deleting the intervening sequence between the switch regions of Cε and Cμ or Cε and Cγ1, respectively. The isotype specificity of CSR is largely determined by cytokines produced by Th cells and dendritic cells. IL-4 signaling through STAT6 is specifically involved in class switch to IgE by inducing germline transcripts of Cε (GLTε) and the expression of activation-induced cytidine deaminase (AID)^24^. Previous studies have demonstrated that PGE2 enhances the expression of GLTε in LPS+IL-4-stimulated B cells in a cAMP-dependent manner, which is presumably responsible for the increased production of IgE^22^. The detailed molecular mechanism, however, remains to be delineated. Furthermore, although pharmacological studies using agonists or antagonists support a role of EP2/EP4 receptors in PGE2 enhancement of IgE production^20^, definitive evidence is missing about the specific receptor(s) involved in this activity.

IgE is thought to have evolved to provide protective immunity against helminth parasites and certain noxious substances^27^,^28^. However, inappropriate IgE responses also constitute a mechanism for allergic diseases such as asthma^29^,^30^. Crosslinking of IgE-FcεRI complexes on mast cells and basophils by allergens leads to the rapid release of inflammatory mediators and subsequent recruitment and activation of inflammatory cells. Besides, IgE also contributes to the long-term pathophysiological changes and tissue remodeling associated with chronic allergic inflammation^30^. In view of the critical role of IgE in asthma, it will be interesting to evaluate the pathological consequence of PGE2 enhancement of IgE production *in vivo*. Over years, a number of studies have investigated the PGE2-mediated effect on various aspects of asthma pathology. Diverse and sometimes opposite effects are revealed^31^. Of note, the role of PGE2 in asthma has never been explored in the context of IgE production.

Using mice with targeted deletion of the gene encoding the EP2 receptor, we analyzed the contribution of EP2-mediated signal in PGE2-enhanced IgE production, the impact of EP2 deficiency on antigen-specific IgE responses *in vivo*, and the relevance of PGE2-regulated IgE production in the development of asthma. In the study of the mechanism underlying PGE2 enhancement of IgE production, we focused on potential modulatory effect of PGE2 signal on STAT6 and NF-κB activation induced by IL-4 and LPS, respectively.

## Results

### Reduced IgE production in EP2-deficient mice

**Figure 1:**
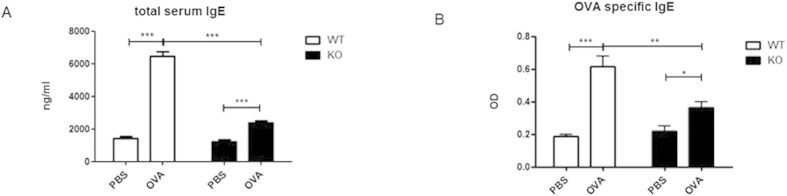
Production of IgE production is impaired in EP2-deficient mice. EP2-deficient mice (KO) and the wild type littermates (WT) were immunized with OVA following a protocol as described in the materials and methods. Control animals received PBS only. Serum levels of total (**A**) and OVA-specific (**B**) IgE were determined by ELISA two days after the last challenge. The concentration of total IgE was calculated using a standard curve obtained with mouse IgE of known concentrations. The relative amount of OVA-specific IgE was depicted by the OD value at a serum dilution of 1:100. Data from 4–6 mice from each group are presented as mean ± SEM. *P < 0.05. **P < 0.01. ***P < 0.005.

PGE2 displays a potent enhancing effect on IgE production by B cells in cultures^19^,^20^,^21^,^22^. But it remains to be determined whether such an action is physiologically relevant. To clarify this issue, we monitored IgE production in mice deficient in EP2, one of the two major PGE2 receptors critical for PGE2-mediated effect on B cells^20^. Serum IgE was present at low but comparable levels in unimmunized EP2 knockout (KO) mice and the wild type littermates (WT). Immunization with OVA induced nearly a 4-fold increase in total serum IgE concentration in WT mice. The KO mice, however, showed only a slight (~80%) increase after the same treatment (Fig. 1A). The difference held true for OVA-specific IgE antibodies. In comparison to WT mice, EP2-deficiency led to a markedly suppressed IgE response to OVA challenge (Fig. 1B). Thus, the EP2-mediated signal is involved in the regulation of IgE response *in vivo*.

### Resistance of EP2-deficient mice to OVA-induced asthma

**Figure 2:**
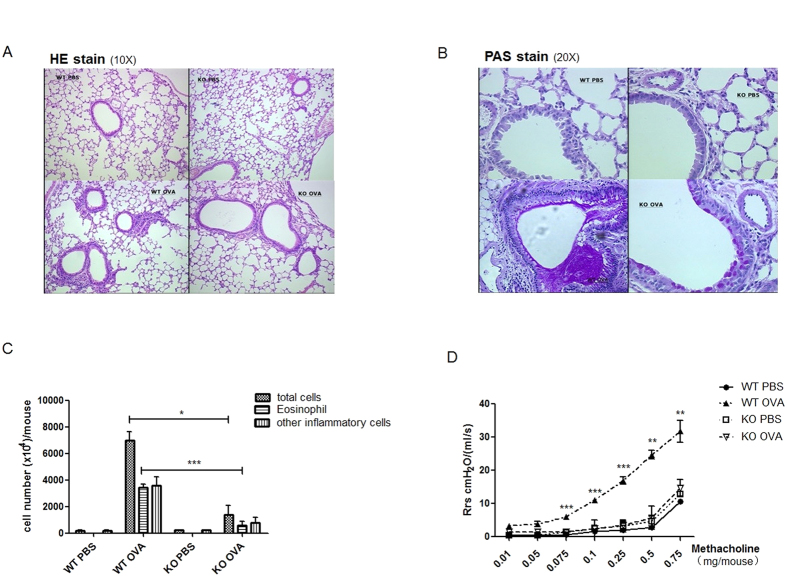
EP2-deficient mice are resistant to OVA-induced asthma. EP2-deficient mice (KO) and the wild type littermates (WT) were sensitized and challenged with OVA as described in the materials and methods. Control animals received PBS only. Pathological analyses were performed two days after the last challenge. (**A,B**) Lung tissues were fixed, sectioned and stained with H&E to reveal lymphocyte infiltration (**A**) or with periodic acid-Schiff (PAS) to show mucus production (**B**). (**C**) BALF was collected and examined for the number of total inflammatory cells and eosinophils. (**D**) The airway hyperresponsiveness in the model animals was assessed by measuring resistance of respiratory system (Rrs) in response to increasing intravenous doses of methacholine chloride. This experiment was repeated three times with 4–6 mice for each group each time. Data are presented as mean ± SEM. *P < 0.05. **P < 0.01. ***P < 0.005.

IgE is believed to be implicated in the pathogenesis of asthma^29^,^30^. The reduced IgE response in EP2-deficient mice prompted us to investigate whether this would lead to altered susceptibility to OVA-induced asthma. First, we examined the infiltration of inflammatory cells and production of mucus in lung tissues following intraperitoneal sensitization and aerosol challenge with OVA. Tissue H&E staining revealed extensive peribronchial and perivascular accumulation of inflammatory cells in OVA-challenged WT mice. The EP2 KO mice, on the other hand, showed much less severe cell infiltration (Fig. 2A). In addition, the overproduction of mucus and goblet cell hyperplasia in the bronchial airway, as shown by PAS staining, was milder in the KO mice than the WT controls (Fig. 2B). The airway inflammation was further assessed by analyzing cells retrieved in BALF. As expected, a large number of inflammatory cells were detected in the airway of OVA-immunized WT mice. In contrast, the total counts, as well as the differential counts of eosinophils, were found to be greatly reduced in BALF from EP2 KO mice (Fig. 2C). Asthma is also featured by airway hyperresponsiveness (AHR). Accordingly, OVA-challenged WT mice exhibited an elevated respiratory resistance in response to methacholine inhalation. Such hyperresponsiveness was greatly suppressed in OVA-challenged EP2 KO mice. In fact, these animals showed a response curve indistinguishable from that of PBS-treated mice (Fig. 2D). These findings support a role of the EP2-mediated signal in the development of asthma, at least in the OVA-induced model.

### Dysregulated IgE production by EP2-deficient B cells

**Figure 3:**
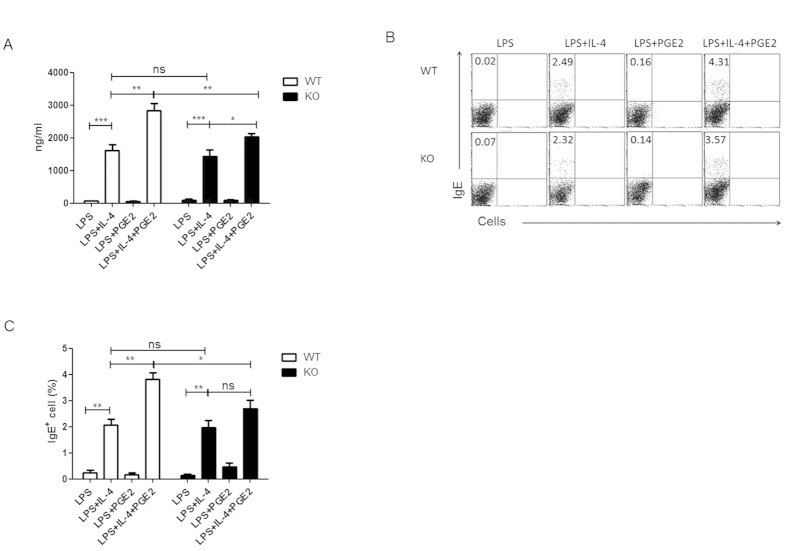
EP2 is largely responsible for PGE2-induced enhancement of IgE production by B cells. Splenic B cells from EP2 knockout (KO) mice or wild type (WT) littermates were stimulated with LPS or LPS+L-4 with or without PGE2. (**A**) IgE concentrations in the culture supernatant at day 7 were measured by ELISA. Data from four independent experiments are presented as mean ± SEM. (**B,C**) The frequency of IgE-secreting B cells at day 4 were detected by intracellular staining of IgE. Experiments were repeated three times. Representative dot plots (**B**) and the percentage of IgE^+^ B cells (**C**) are shown. *P < 0.05. **P < 0.01. ***P < 0.005. ns, not significant.

We next sought to determine whether the reduced serum levels of IgE in EP2 KO mice was due to an intrinsic defect in B cells. To this end, splenic B cells were purified from the KO and WT mice and cultured in the presence of LPS or LPS+IL-4 with or without the addition of PGE2. Seven days later, the culture supernatant was collected and assayed for the IgE level using ELISA. Consistent with previous reports^20^,^22^, significant amounts of IgE were only detected in cultures stimulated with LPS+IL-4 and the addition of PGE2 almost doubled IgE production by wild type B cells. EP2-deficient B cells produced comparable amounts of IgE under stimulation with LPS+IL-4. The PGE2 enhancing effect, however, was markedly attenuated, with only 40% increase over that without PGE2 (Fig. 3A). Furthermore, we measured the frequencies of IgE-producing cells in the cultures at day 4 by intracellular staining and flow cytometry. Upon stimulation with LPS+IL-4, the wild type and EP2-deficient B cells gave rise to an equal percentage of IgE-producing cells. While the frequency was further increased in wild type B cell cultures with the addition of PGE2, IgE^+^ cells generated from EP2-deficient B cells remained at similar levels in the presence or absence of PGE2 (Fig. 3B,C). Therefore, the EP2-mediated signal is critically involved in the regulation IgE production by B cells and its deficiency renders B cells less responsive to PGE2 stimulation in IgE production.

### Impaired IgE class switch as a major contributor to the compromised IgE production by EP2-deficient B cells

**Figure 4:**
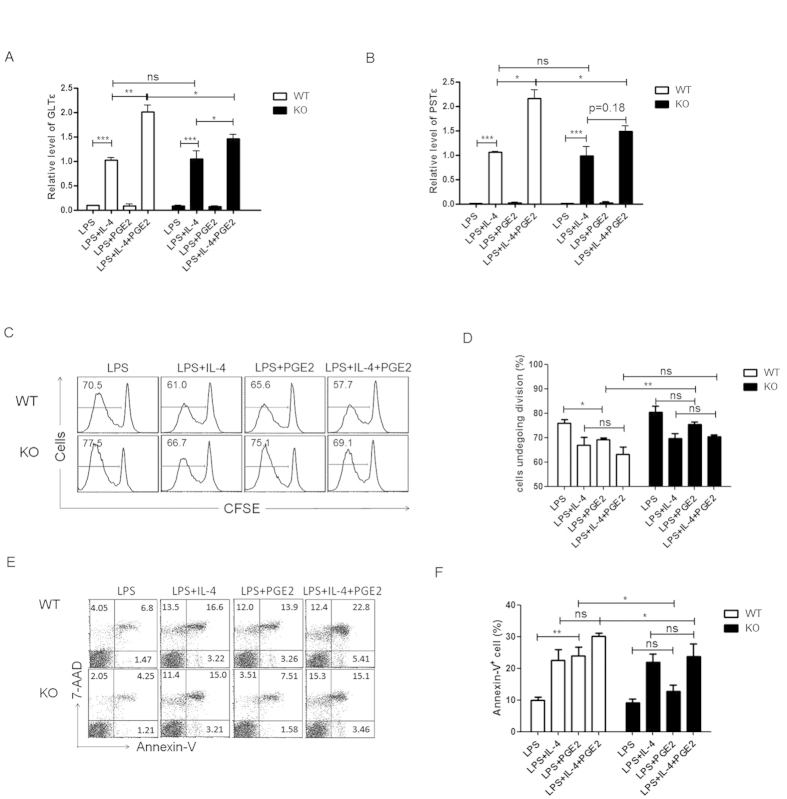
EP2-deficiency impairs IgE production mainly by affecting IgE class switching. Splenic B cells from EP2 knockout (KO) mice or wild type (WT) littermates were stimulated with LPS or LPS+IL-4 in the presence or absence of PGE2. (**A,B**) RNA was prepared from day 3 and day 5 cultures and examined for germline transcripts ε (GLTε) (**A**) and post switch transcripts ε (PSTε) (**B**), respectively. The expression levels were calibrated against that in LPS+IL-4-treated wild type B cells. Data from at least four independent experiments (each with duplicates) are presented as mean ± SEM. (**C,D**) Cell proliferation was measured by CSFE dilution. Representative histograms (**C**) and the percentage of cells undergoing at least one division (**D**) are shown. (**E,F**) Cell apoptosis was analyzed by Annexin-V and 7-AAD staining. Representative dot plots (**E**) and the percentage of Annexin-V^+^ cells (**F**) are shown. Experiments were repeated at least three times. Data are presented as mean ± SEM. *P < 0.05. **P < 0.01. ***P < 0.005. ns, not significant.

The generation of different classes of Ig is achieved through class switch recombination (CSR)^23^,^26^. The isotype specificity of CSR is critically dependent on the transcriptional activation of the locus encoding the constant region of a particular Ig class (Cε in the case of IgE), which leads to the generation of “sterile” germline transcripts. To explore the role of the EP2-mediated signal in IgE class switch, we measured GLTε levels in B cells cultured under different conditions for 3 days using quantitative PCR. As reported by Roper *et al.*^22^, LPS+IL-4 strongly activated germline transcription of the Cε locus, which was further enhanced in the presence of PGE2. Although EP2-deficiency had no significant influence on GLTε expression induced by LPS+IL-4, it greatly reduced the enhancing effect of PGE2 (Fig. 4A). We also compared the levels of post-switch mature IgE transcripts in wild type and EP2-deficient B cells in day 5 cultures. Similar to what was observed for GLTε, PGE2 enhancement of PSTε expression was much attenuated in the absence of EP2 (Fig. 4B). Thus, EP2 is required for PGE2 enhancement of IgE class switch.

We further addressed the possibility that EP2-mediated signal may affect other aspects of B cell activation, such as cell proliferation and survival, which in turn contributes to the reduced IgE production. As indicated by CFSE dilution, PGE2 caused a slight but significant inhibition of LPS-induced proliferation of wild type B cells. Such an effect was dependent on EP2 as it was not observed with EP2-deficient B cells (Fig. 4C,D). As much as cell viability is concerned, EP2 deficiency actually improved the survival of B cells stimulated by LPS or LPS+IL-4 (Fig. 4E,F).

Taken together, these results suggest that the EP2-mediated signal promotes IgE production most likely through enhancement of IgE class switch and development of IgE-secreting B cells.

### Modulation of the IL-4-STAT6 signaling by EP2-mediated signal

**Figure 5:**
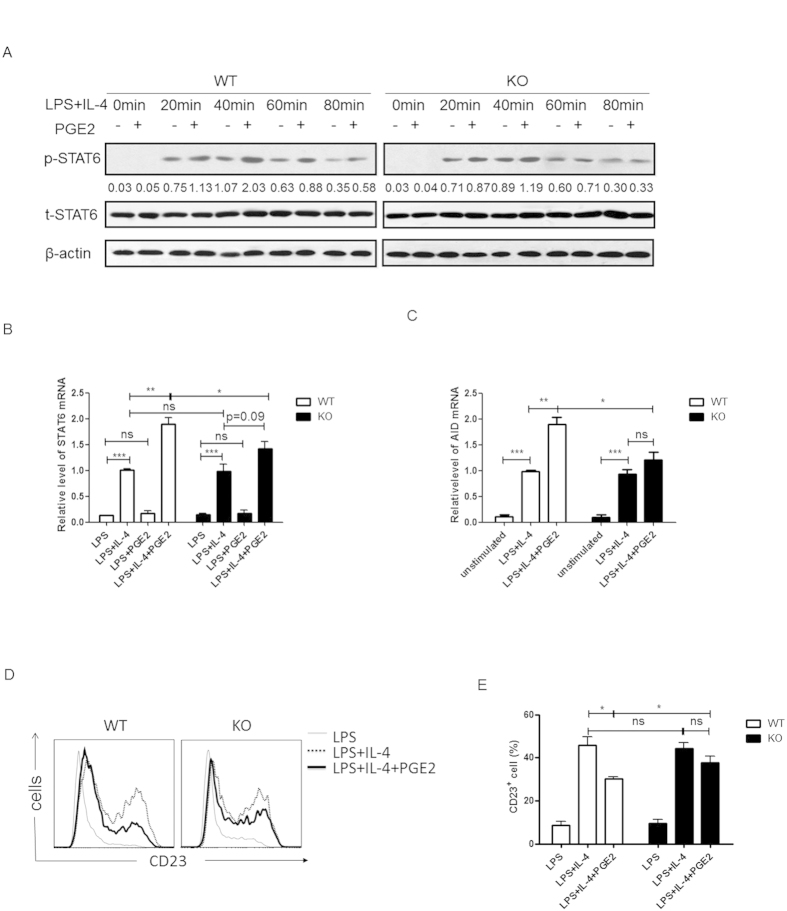
EP2-mediated signal enhances STAT6 phosphorylation and AID expression but inhibits CD23 expression. Splenic B cells from EP2 knockout (KO) mice or wild type (WT) littermates were stimulated with LPS or LPS+IL-4 with or without PGE2. (**A**) Cells were harvested at 0, 20, 40, 60 and 80 min after stimulation. Phosphorylated and total STAT6 was detected by Western blotting. The cropped gels have been run under the same experimental conditions. Representative blots are shown of three independent experiments. The numbers indicate the ratio of phosphorylated versus total STAT6 under various conditions. (**B,C**) RNA was prepared from day 3 cultures and examined for STAT6 (**C**) and AID mRNA expression by quantitative PCR. The expression levels were calibrated against that in LPS+IL-4-treated wild type B cells. (**D,E**) Surface expression of CD23 was determined by flow cytometry at day 3. Representative histogram (**D**) and the percentage of CD23^+^ cells (**E**) are shown. Data from at least four independent experiments are presented as mean ± SEM. *P < 0.05. **P < 0.01. ***P < 0.005. ns, not significant.

The mechanism by which PGE2 enhances IgE class switch remains elusive. Among the multiple factors involved in the regulation of IgE class switch, IL-4-STAT6 signaling is of particular importance as the levels of IgE are dramatically reduced in IL-4- and STAT6-deficient mice^32^,^33^. So we focused our analysis on the modulatory effect of PGE2 on this signaling pathway. Purified splenic B cells were stimulated with LPS or LPS+IL-4 with or without the addition of PGE2 and the phosphorylation of STAT6 was examined by Western blotting. As shown in Fig. 5A, PGE2 treatment substantially enhanced the phosphorylation of STAT6 induced by IL-4 in wild type B cells. This effect, however, was barely observed in EP2-deficient B cells, suggesting that PGE2 exerts such an activity mainly through EP2. In addition to enhanced STAT6 activation, STAT6 mRNA levels were also found to be up-regulated in B cell cultures harvested 3 days after exposure to PGE2 (Fig. 5B). This is most likely the secondary effect of an increased STAT6 activity, which is known to promote its own transcription^34^.

In B cells, STAT6 regulates the expression of a wide array of genes, some of which are critically involved in IgE production^35^. In addition to a well established role in the activation of germline transcription of the Cε locus, STAT6 induces the expression of activation-induced cytidine deaminase (AID), which is essential for CSR. Along with the enhanced phosphorylation of STAT6, PGE2 treatment led to approximately a 2-fold increase of AID mRNA expression in LPS+IL-4-treated wild type B cells, whereas AID expression in EP2-deficient B cells was not significantly altered with or without PGE2 (Fig. 5C).

CD23, a negative regulator of IgE response, is another target gene of STAT6. Following IL-4 stimulation, STAT6 activity promotes the expression of CD23, which presumably constitutes a negative feedback mechanism for IL-4-induced IgE production^24^,^36^. Given the enhanced STAT6 phosphorylation, one might expect an elevated level of CD23 in PGE2-treated cells. Contradictory to this expectation but consistent with previous reports^20^, surface expression of CD23 was actually found to be down-regulated in the presence of PGE2 compared to cells stimulated with LPS+IL-4 only. Again, EP2-deficient B cells were less responsive to the inhibitory effect of PGE2 (Fig. 5D,E). Although the mechanism is unclear, this phenomenon is particularly interesting in that PGE2 is able to break the negative feedback loop while maintaining a stimulatory effect on STAT6 activation.

In addition to STAT6, the classical pathway to IgE switching involves NF-κB activity, for which both CD40 and LPS are potent inducers^26^. As such, we further investigated the impact of PGE2 signal on NF-κB activation in LPS+IL-4-stimulated B cells. While phosphorylation of p65 was readily detectable in wild type and EP2-deficient B cells treated with LPS+IL-4, their levels remained unaltered in the presence or absence of PGE2 (Suppl. Fig. 1). Therefore, the impact of PGE2 on IgE production seems to be largely attributable to enhanced STAT6 phosphorylation.

## Discussion

The present study explored the role of EP2-mediated signal in the regulation of IgE production and its pathological relevance in asthma. The major findings are briefly summarized as follows: 1) the EP2 receptor is required for PGE2-enhanced IgE production by B cells; 2) EP2-mediated signal increases IgE production by facilitating IL-4-induced STAT6 activation and transcription of downstream targets critical for IgE class switching; 3) PGE2 signaling through EP2 promotes antigen-specific IgE responses *in vivo* and contributes to the development of OVA-induced asthma.

Previous analyses with EP-selective agonists suggest that both EP2 and EP4 receptors contribute to PGE-induced effect on B cells^20^. The present study provides genetic evidence for a critical role of EP2 in PGE2-regulated production of IgE. Normally, addition of PGE2 to B cell cultures under stimulation with IL4+LPS promotes STAT6 phosphorylation, and IgE class switching and production. The synergism, however, was markedly diminished in the absence of EP2. Nevertheless, a minimal B cell response to PGE2 was still maintained without EP2, indicating that other receptor subtypes, such as EP4, might also play a role in PGE2-enhanced IgE production.

IgE class switching is critically dependent on the transcription factors NF-κB and STAT6. Upon activation by CD40 ligation or LPS stimulation, NF-κB synergizes with STAT6, which is principally activated by IL-4 or IL-13, to induce Cε germline transcription and AID expression, two events essential for CSR to IgE^26^. To explore the molecular mechanism underlying PGE2 enhancement of IgE production, we examined the impact of PGE2 signaling on the activation of NF-κB and STAT6 in B cells stimulated with LPS+IL-4. NF-κB p65 phosphorylation was not altered with the addition of PGE2. In contrast, PGE2 led to a substantial increase in the level of phosphorylated STAT6. As two major targets downstream of STAT6^37^,^38^, GLTε and AID mRNA expression was found to be upregulated along with the hyper-activation of STAT6. In addition to the enhancing effect on STAT6 activation, prolonged treatment with PGE2 also caused an upregulation of STAT6 mRNA expression. This appears to be consistent with a gene expression profiling analysis by Schroder *et al.*, in which STAT6 is suggested having a self-promoting potential on its own transcription^34^. Such a positive feedback may provide a mechanism to amplify the effect of PGE2. IL-4-induced STAT6 activity not only promotes class switch to IgE, but also induces CD23 expression, which presumably provides a feedback mechanism to prevent an excessive IgE response^24^,^36^. Intriguingly, despite the enhanced activation of STAT6, surface expression of CD23 was down-regulated in the presence of PGE2. Similarly, PGE2 has been reported to inhibit class II MHC expression induced by IL-4-STAT6 signaling^20^,^39^. Therefore, certain PGE2 actions are apparently independent of STAT6. The enhanced class switching to IgE and the concomitant down regulation of CD23 expression make PGE2 a particularly potent inducer of IgE production.

Although the enhancing effect of PGE2 on IgE production was well illustrated with purified B *in vitro*^19^,^20^,^21^,^22^, data are fragmentary and controversial concerning its role in IgE response *in vivo*. Several studies monitored IgE levels in mice deficient for PGE2 synthesis^40^,^41^,^42^. Church *et al.* reported that deficiencies in neither Cox-1, Cox-2 nor mPGES1 affected the serum levels of IgE after immunization with OVA^40^. Comparable levels of IgE were also documented in mPGES-1 knockout and wild type mice in a house dust mite antigen (Der f)-induced allergic model^41^. Still another study, however, demonstrated a significant increase of total IgE in the BALF from OVA-allergic Cox-1- and Cox-2-deficient mice while it was barely detectable in wild type controls^42^. More recently Zaslona and colleagues directly explored the regulatory role of EP2-mediated signal in IgE production in the context of OVA-induced asthma. In contrast to the results presented here, they showed that EP2 deficiency led to elevated serum IgE levels, which, they proposed, was primarily the consequence of enhanced T cell responses in the absence of EP2-mediated inhibition^43^. The exact reason for the discrepancy in unclear but may be partly related to the different protocols of immunization. In our hands, the mice were sensitized by three weekly intraperitoneal injections of OVA, which led to a fully developed IgE response with a serum concentration up to 1500 ng/ml. In their study, mice were subject to analyses one week after a single injection of OVA. The latter protocol appeared to be less effective for the induction of IgE production as suggested by the low levels of serum IgE^43^. Alternatively, the response may still be at its early stage. Both possibilities could affect the regulatory role of PGE2-EP2 signaling.

The role of PGE2 in the pathogenesis of asthma has been intensively studied. The majority of early studies indicate a beneficial effect^31^. While inhalation of PGE2 or its analogs ameliorates allergen-induced airway inflammation in asthmatic patients and animal models^44^,^45^, treatment of mice with COX inhibitors augments allergic inflammation and airway hyperresponsiveness^46^,^47^. Consistent with these findings, mice deficient in either COX-1 or COX-2 were found to develop a much severe disease in OVA-induced asthma models^40^,^42^. To clarify the specific role of PGE2 in the protection, Lundequist *et al.* examined disease development in mPGES-1-deficient mice using a Der f-induced allergic model^41^. While reduced PGE2 synthesis had no significant impact on allergen sensitization and generation of effector T cells, marked remodeling of the pulmonary vasculature occurred in mPGES-1-deficient mice during chronic exposure to allergen. The hypothesis of PGE2-mediated protection was reinforced by observations of exaggerated allergic responses in mice lacking EP3^48^ or EP2 receptors^43^. The EP3 signal is believed to act on epithelial cells at a step subsequent to IgE, probably by inhibiting synthesis of chemokines like CCL11 and CCL17^48^. The EP2 signal, on the other hand, is thought to dampen allergic responses by targeting CD4^+^ T cells at the sensitization stage^43^.

Opposite to the heightened allergic responses reported by Zaslona *et al.*^43^, our results demonstrated that EP2-deficiency attenuated lung inflammation in OVA-induced asthma. To some extent, this is reminiscent of the controversy over the role of mPGES-1 in airway inflammation. While conferring protection against vascular remodeling induced by chronic inflammation^41^, mPGES-1 and its product, PGE2, promote acute allergic responses in OVA-induced asthma models^40^. The conflicting results about EP2 signal in asthma may also reflect differences in experimental designs. In the study by Zaslona et el., mice were analyzed one week after a single injection of OVA. In such a setting, the allergic response seemed to be underdeveloped as indicated by the poor recruitment of eosinophils and other inflammatory cells, which were 10~30-folds lower than what was obtained in our studies^43^. While this design may be suitable for analysis of the role of EP2 signal in T cells during early sensitization, it prevents full manifestation of EP2-mediated actions on other cell type, including B cells, in late phases.

In summary, the present study shows that PGE2-enhanced IgE production by B cells is mainly mediated by EP2, possibly through synergistic activation of STAT6. Loss of EP2 leads to a reduced IgE response to allergens and attenuated airway inflammation in the OVA-induced asthma model. These data not only reveal an important regulatory role of the PGE2-EP2-STAT6 signaling pathway in IgE response but also underscore the challenge in the development of therapeutic strategy targeting PGE2 and its receptor in allergic diseases.

## Methods and Materials

The methods were carried out in accordance with the approved guidelines of Peking University Health Science Center.

### Experimental animals

The EP2 knockout mice was generated as previously described^49^ and backcrossed onto the C57BL/6J background for 6–8 generations. Genotyping was performed using PCR^49^. Experiments were carried out on female animals between 6–8 weeks of age with the wild type littermates as controls. All care and handling of animals were performed according to the standard guidelines for the care and use of experiment animals in Peking University Health Science Center.

### OVA-induced asthma model

Mice were sensitized by intraperitoneal injections of 20μg of OVA (Sigma-Aldrich, St. Louis, MO) emulsified in 2 mg aluminum hydroxide (Pierce Chemical, Rockford, IL) in a total volume of 200μl on days 1, 8, 15, and challenged with an aerosol instillation of 1% OVA in PBS for 30 min on days 19, 21, 23. Control animals received PBS only. Two days after the final challenge, lung mechanics were measured, and the lung, serum and bronchoalveolar lavage fluid (BALF) were collected for further analysis.

### Measurement of airway hyperresponsiveness

Hyperresponsiveness of airways was measured by placing mice in a whole-body plethysmograph (model AniRes2005; Beatlab Technology Co., Beijing, China)^50^. Briefly, 48 hours after final challenge, mice were anaesthetized intraperitoneally with 0.4% pentobarbital sodium (90 mg/kg), tracheostomized and connected to the ventilator. Mechanical ventilation was carried out by setting the ratio of expiration to inspiration at 15:10 and respiratory rate at 90 per minute. Dynamic airway pressure (ΔP) and volume of chamber (ΔV) were recorded after administration of increasing doses (0.01, 0.05, 0.075, 0.1, 0.25, 0.5, 0.75 mg/mouse) of methacholine chloride through the indwelling needle in external jugular vena, and the resistance of respiratory system (Rrs) was calculated as Rrs = ΔP/(ΔV/ΔT).

### Inflammatory cell counts in BALF

Immediately after intraperitoneal injection with an overdose of pentobarbital (50 mg/kg), 0.6 ml of ice-cold PBS was instilled into the lung via the tracheal cannula and recovered by gentle manual aspiration. BALF collected from 3 repeats (total volume 1.8 ml) was pooled and centrifuged. The total number of inflammatory cells were counted using cell counting chamber and the differential cell counts were obtained following staining with Diff-Quik Stain reagent (Yuanmu Biotechnologies, Shanghai, China).

### Lung tissue histopathology

The lung tissue was fixed in 10% neutral buffered formalin for 24 h, embedded in paraffin, sectioned at 4 μm thickness, and stained with hematoxylin and eosin (H&E) to examine cell infiltration or with periodic acid-Schiff (PAS) to measure mucus production.

### Flow cytometry and cell sorting

Single cell suspensions were prepared from the spleen and the erythrocytes were depleted with the ACK lysis buffer. For surface staining, cells were incubated for 25 min at 4 °C with fluorescent-labeled monoclonal Ab specific for mouse CD23, B220, CD4, CD44 and/or CD62L (BD Biosciences, San Jose, CA). To detect CD23 expression, 0.05% EDTA was included in the staining buffer to suppress IgE binding. For intracellular staining, cells were first fixed and permeabilized with Cytofix/Cytoperm (BD Biosciences) and then incubated with PE-conjugated anti-IgE (Biolegend, San Diego, CA). Appropriate isotype-matched Abs were utilized for compensation adjustment. Flow cytometric analysis was performed on FACSCalibur using CellQuest software (BD Biosciences). B220^+^ B cells were sorted using FACSAria (BD Biosciences) with a purity > 98%.

### B cell culture

Purified B cells were cultured at 1 × 10^6^ cells/ml in Opti-MEM (Invitrogen, Carlsbad, CA) supplemented with 10% fetal calf serum (FCS). To induce IgE production, cells were stimulated with LPS (20 μg/ml, Sigma-Aldrich), or LPS+IL-4 (50 ng/ml, R&D Systems, Minneapolis, MN) for 3–7 days in the presence or absence of PGE2 (Cayman Chemical, Ann Arbor, MI). Different concentrations of PGE2, ranging from 10^−10^ M to 10^−6^ M, were tested in preliminary experiments. PGE2 at 10^−8^ M resulted in significant enhancement of IgE production with minimal effects on cell survival and was therefore used for subsequent experiments.

### Proliferation assay

For proliferation assay, 5 × 10^6^ B cells were incubated at room temperature for 5 min in 1 ml of PBS containing 5 μM carboxy fluorescein diacetate succinimide ester (CFSE, Invitrogen). Cells were washed twice to remove free dye before being put into culture under various conditions as described above. After culture for 3 days, cells were monitored for CFSE dilution using flow cytometry.

### Apoptosis assay

Purified B cells were cultured under various conditions for 3 days. Cell apoptosis was determined by staining with FITC-coupled Annexin V (Biosea Biotechnology Co. Ltd., Beijing, China) and 7-amino-actinomycin D (7-AAD, BD Boisciences), followed by analysis on a FACSCalibur.

### Enzyme-linked immunosorbent assay (ELISA)

Total IgE in the serum and culture supernatant was measured using OptEIA™ Mouse IgE ELISA Set (BD Biosciences) according to the manufacturer’s instruction. The concentration of IgE was calculated using a standard curve obtained with mouse IgE of known concentrations. To measure serum levels of OVA-specific IgE, serially diluted sera was added into 96-well plates pre-coated with anti-IgE (2 μg/ml, BD Biosciences), followed by incubation with biotinylated OVA (1.25 μg/ml, Chondrex, Redmond, WA). The bound biotinylated OVA was detected with horseradish peroxidase (HRP)-conjugated streptavidin (R&D Systems) using tetramethylbenzidine as substrate (Sigma-Aldrich). The absorbance was read at 450/490 nm.

### Quantitative RT-PCR

Total RNA from cultured B cells was isolated with Trizol Reagent (Invitrogen) and was used to make cDNA oligo-dT primers and the Reverse Transcription System (Promega, Madison, WI). For quantitative PCR, iQ SYBR Green Supermix (Bio-Rad Laboratories, Hercules, CA) was used according to the manufacturer’s instructions. The amplification was performed on an iCycler (Bio-Rad Laboratories). The quantification was based on ΔCT and calibrated to the level in LPS+IL-4-treated wild type B cells. GAPDH was used as an internal control. Primers were used as follow: *Aid* forward, 5′-TGCTACGTGGTGAAGAGGAG-3′, reverse, 5′-TCCCAGTCTGAGATGTAGCG-3′; GLTε forward, 5′-GCACAGGGGGCAGAAGAT-3′, reverse, 5′-CCAGGGTCATGGAAGCAGTG-3′; PSTε forward, 5′-TTGGACTACTGGGGTCAAGG-3′, reverse, 5′-CAGTGCCTTTACAGGGCTTC-3′; *Stat6* forward, 5′-TGAGGTGGGGACCAGCCGG-3′, reverse, 5′-GTGACCAGGACACACAGCGG-3′; *Gapdh* forward, 5′-TTCACCACCATGGAGAAGGC-3′, reverse, 5′-GGCATGGACTGTGGTCATGA-3′.

### Western Blotting

B cell cultured under various conditions were harvested and cell lysates were prepared in lysis buffer (20 mM Tris (pH 8.0), 137 mM NaCl, 5 mM EDTA, 10% glycerol, 1% Triton X-100, 1 mM PMSF, 1 mM aprotinin, 1 mM leupeptin, 1 mM EGTA, 1 mM Na3VO4, 1 mM tetrasodium pyrophosphate, and 10 mM NaF). The lysate was resolved on a 12% reducing SDS-polyacrylamide gels and transferred to a polyvinylidene difluoride membrane. After blocking with Tris-buffered saline (pH 7.4) containing 5% dried skimmed milk, the membrane was incubated with anti-STAT6 (Cell Signaling Technology, Beverly, MA), anti-phospho-STAT6 (Millipore Technology, Billerica, MA), anti-NF-κB p65 (Cell Signaling Technology), anti-phospho-p65 (Cell Signaling Technology), or anti-β-actin (Cell Signaling Technology), followed by probing with HRP-conjugated anti-rabbit antibody (Sigma Aldrich). The immunoreactive bands were detected by chemiluminescence with ECL detection reagents (Life Technologies, Grand Island, NY, USA).

### Statistics

All experiments were repeated at least three times, with 4–6 mice for each group each time. Data are presented as mean ± the standard error of the mean (SEM) unless otherwise specified. For statistical analysis, unpaired Student’s t test was performed using GraphPad Prism software (GraphPad Software, La Jolla, CA). A *P* value less than 0.05 was considered as statistically significant.

## Additional Information

**How to cite this article**: Gao, Y. *et al.* Prostaglandins E2 signal mediated by receptor subtype EP2 promotes IgE production in vivo and contributes to asthma development. *Sci. Rep.* **6**, 20505; doi: 10.1038/srep20505 (2016).

## Supplementary Material

Supplementary Information
