# Supplementary material for: Prostaglandins E2 signal mediated by receptor subtype EP2 promotes IgE production in vivo and contributes to asthma development
Source: Sci Rep. 2016 Feb 8;6:20505. doi: 10.1038/srep20505 (PMC4744937; doi:10.1038/srep20505)

**TITLE PAGE**

**Prostaglandins E2 signal mediated by receptor subtype EP2 promotes IgE production in vivo and contributes to asthma development**

Yuhan Gao^a*^, Chunyan Zhao^a*^, Wei Wang^a^, Rong Jin^a^, Qian Li^a^, Qing Ge^a^, Youfei Guan^b＃^and Yu Zhang^ac#^

^a^Department of Immunology, and Key Laboratory of Medical Immunology of Ministry of Public Health, Peking University Health Science Center, Beijing, China

^b^Advanced Institute for Medical Sciences, Dalian Medical University, Dalian, China

^c^State Key Laboratory of Natural and Biomimetic Drugs, Peking University, Beijing, China

*These authors contribute equally to this work.

#To whom correspondence should be addressed at:

Yu Zhang, Department of Immunology, Peking University Health Science Center, 38 Xue Yuan Road, Beijing, 100191, China, Tel: 0086-10-82802593, Fax: 0086-10-82801436, E-mail: zhangyu007@bjmu.edu.cn

Youfei Guan, Advanced Institute for Medical Sciences, Dalian Medical University, 9 West Section Lvshun South Road, Dalian, Liaoning 116044, China, Tel: 0086-411-86110021, Fax: 0086-411-86110021, E-mail: guanyf@dmu.edu.cn


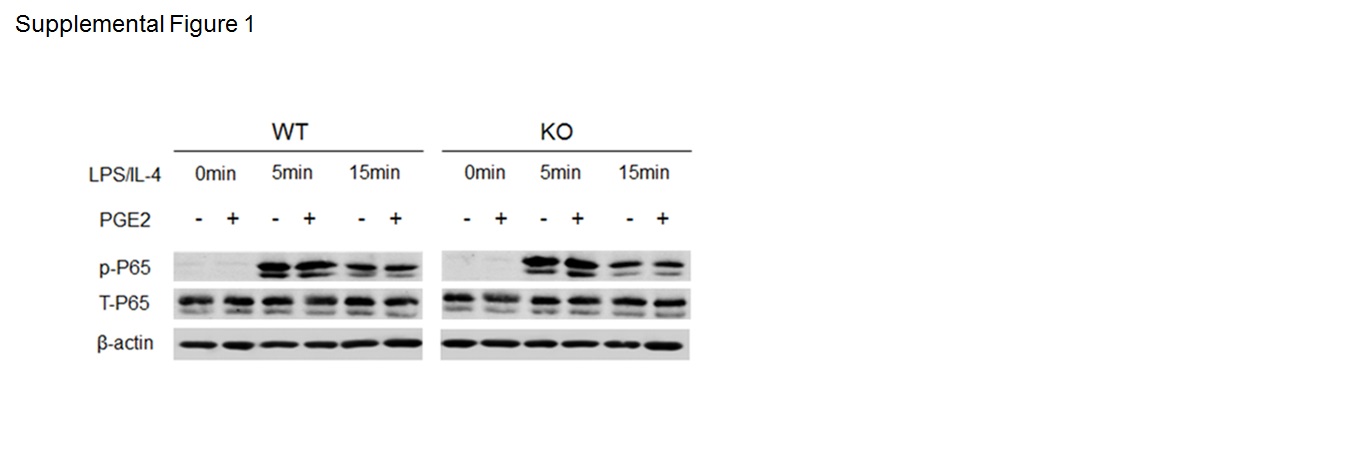

Supplement: Supplementary Information [file srep20505-s1.doc]
